# ARGformer: learning on ancestral recombination graphs with transformers

**DOI:** 10.1093/bioinformatics/btag438

**Published:** 2026-08-21

**Authors:** David Bonet, Cole Shanks, Marçal Comajoan Cara, Jordi Abante, Alexander G Ioannidis

**Affiliations:** Department of Biomedical Data Science, Stanford University, Stanford, CA, United States; Genomics Institute, University of California, Santa Cruz, Santa Cruz, CA, United States; Department of Biomedical Sciences, Universitat de Barcelona, Barcelona, Catalonia, Spain; Genomics Institute, University of California, Santa Cruz, Santa Cruz, CA, United States; Genomics Institute, University of California, Santa Cruz, Santa Cruz, CA, United States; Department of Electrical Engineering and Computer Science, University of California, Berkeley, Berkeley, CA, United States; Department of Biomedical Sciences, Universitat de Barcelona, Barcelona, Catalonia, Spain; Department of Biomedical Data Science, Stanford University, Stanford, CA, United States; Genomics Institute, University of California, Santa Cruz, Santa Cruz, CA, United States

## Abstract

**Motivation:**

Recent advances in inference of the ancestral recombination graph (ARG), which describes how segments of chromosomes trace back through recombination and shared lineages, have made it possible to reconstruct genome-wide genealogies for large cohorts, but it remains difficult to summarize and use this information for population genetic analyses.

**Results:**

We present ARGformer, an encoder-only transformer that learns context-dependent embeddings with a self-supervised masked objective finetuned with contrastive learning for downstream retrieval tasks. We train ARGformer on genealogies from coalescent simulations and on genealogies inferred from ancient and present-day *Homo sapiens* genomes. Using only these learned embeddings, without access to genotype matrices, ARGformer captures patterns of global population structure and supports ancestry inference through clustering and nearest-neighbor retrieval. On genealogies that include archaic hominins, ARGformer can highlight Denisovan-derived segments in Oceanian genomes and reveals Oceanian-like ancestry in South American Indigenous populations.

**Availability and Implementation:**

ARGformer is available at https://github.com/AI-sandbox/ARGformer.

## 1 Introduction

The high dimensionality of genomic data, especially in large population-scale sequencing studies such as national biobanks, has motivated the development of a variety of methods for visualizing population structure (Bycroft *et al.* 2018). The most popular of these is principal component analysis (PCA) (Patterson *et al.* 2006), as well as uniform manifold approximation and projection (UMAP) (Diaz-Papkovich *et al.* 2019). In the context of population genetics, these dimensionality reduction techniques provide insight into population structure and relatedness. Deep learning has been applied for visualizing population structure in the form of variational autoencoders (VAEs) trained on SNP genotypes. VAEs produce low-dimensional latent embeddings that capture nonlinear population structure and often preserve global geometry better than t-SNE or UMAP applied directly to SNP genotypes (Battey *et al.* 2021, Geleta *et al.* 2026). Other approaches include MAPS, which estimates time-stratified migration-rate surfaces from identity-by-descent (IBD) tracts, providing temporal visualization of structure (Al-Asadi *et al.* 2019). Collectively, these methods provide powerful low-dimensional summaries of genetic data, but they operate directly on genotypes or derived summaries, rather than on the underlying genealogies that generate those genotypes.

Patterns of allele sharing and population structure are embedded in the history of genealogies under the coalescent with recombination (Hudson 1983, Griffiths and Marjoram 1997). The ancestral recombination graph (ARG) provides a unifying representation of this history by encoding the collection of local genealogical trees along the genome together with recombination events that rearrange ancestry through time (Griffiths and Marjoram 1997, Lewanski *et al.* 2024, Nielsen *et al.* 2025). Genealogical interpretations of principal component analysis make this link explicit by viewing genotype structure as a projection of the underlying genealogy (McVean 2009). In the presence of recombination, the ARG encodes information about population size changes, migration and admixture, recombination and mutation processes, linkage disequilibrium, and identity by descent (Nielsen *et al.* 2025). In principle, methods that learn directly from ARGs have the potential to unify many classical population genetic analyses within a common representation.

Recent methods can now infer genome-wide genealogies at scale for large cohorts, demonstrating the utility of inferred ARGs for genome-wide association studies and population structure analyses (Rasmussen *et al.* 2014, Kelleher *et al.* 2019, Speidel *et al.* 2019, Zhang *et al.* 2023, Gunnarsson *et al.* 2024, Deng *et al.* 2025, Nielsen *et al.* 2025). However, there is not yet a standard, scalable self-supervised framework for representation learning directly on genome-wide genealogies or ARG-derived structures analogous to the way that VAEs have been applied to genotypes. This could be related to the challenges associated with such a task. For example, it is not immediately clear how to input an ARG into a deep learning model in the way that VAEs can process genotype data. In addition, the complete ARG can be an ultra-large graph even for a modest cohort, and the marginal coalescent trees also become very large graphs for medium to large scale cohorts. More generally, although deep learning has been used for population genetic inference and for learning features from sequence data (Sheehan and Song 2016, Schrider and Kern 2018, Flagel *et al.* 2019), there is no standard framework for self-supervised representation learning directly on genome-wide genealogies. Recently, transformer architectures have begun to be applied to population genetics. A recent example is cxt, a decoder-only transformer that autoregressively predicts pairwise coalescence times from local mutational context (Korfmann *et al.* 2025). This line of work shows that transformers can be effective for genealogically informed inference from sequence-derived inputs. ARGformer addresses a complementary problem: rather than inferring coalescence from mutational context, it learns representations directly from inferred genealogies, enabling downstream tasks such as visualization, clustering, and retrieval on ARG-derived structures.

An architecture for efficiently encoding sequences in natural language processing has been encoder-only transformers, and in particular Bidirectional Encoder Representations from transformers (BERT) models (Devlin *et al.* 2019). Masked language modeling objectives allow such models to learn rich, context-dependent representations in a fully self-supervised fashion, and recent work has introduced more efficient architectures that scale to large training corpora (Warner *et al.* 2025). Encoder-only transformers and masking objectives have also been successfully transferred to a wide range of biological sequence and graph domains, where they provide general-purpose embeddings for downstream tasks (Ji *et al.* 2021, Rives *et al.* 2021). More broadly, recent theoretical and empirical work has shown that pure transformer architectures, with suitable tokenization schemes, can act as powerful and competitive graph learners, often rivaling or surpassing message passing graph neural networks (Kim *et al.* 2022).

Here we explore an encoder-only model for representing coalescent events in the ARG, which we call ARGformer. The model encodes, for each extant haplotype, the path from the leaf to the root as a token sequence, exploiting shared coalescent events across paths. ARGformer learns context-dependent embeddings that recapitulate global population structure, capture local genealogical relationships, and support downstream local ancestry analyses via clustering or nearest-neighbor retrieval in embedding space. The advantage of ARGformer is that it compresses the massive, ultra-high-dimensional structure of the ARG into a dense, accessible latent space, enabling downstream methods to query complex coalescent histories without directly parsing the raw graph topology. Conceptually, our approach is related to genealogical interpretations of principal component analysis in population genetics, which view genotype structure as a projection of the underlying genealogy (McVean 2009), but ARGformer operates directly on the topological structure of inferred genealogies, leveraging the evolutionary history already captured by the ARG rather than attempting to learn these relationships from raw genotype matrices.

In applications to both simulated genealogies and ARGs inferred from ancient and present-day *Homo sapiens* genomes, we show that ARGformer embeddings alone, without access to genotype matrices, recover patterns of global population structure and support ancestry inference via clustering and nearest-neighbor retrieval. On ARGs that include archaic hominin genomes, ARGformer embeddings highlight Denisovan-like segments in Oceanian genomes, consistent with previous observations of Denisovan introgression in Papuans (Jacobs *et al.* 2019, Vespasiani *et al.* 2022), and reveal Oceanian-like ancestry patterns present in some Indigenous South American populations that are not evident in the other Indigenous American groups. This echoes previously observed Australasian-related ancestry in Amazonian groups (Skoglund *et al.* 2015, Castro e Silva *et al.* 2021). These examples illustrate how local genealogies along the genome encode complex demographic and admixture histories, and how representation learning on ARGs can make this information accessible for visualization and downstream analyses.

## 2 Materials and methods

### 2.1 BERT model for encoding paths in ancestral recombination graphs

There are many possibilities for encoding ARGs with a transformer. Rather than encoding an entire ARG or full marginal coalescent trees, we encode the coalescent events along leaf-to-root paths. Because different paths share internal ancestral nodes and adjacent marginal trees share substantial structure along the genome, this representation retains substantial genealogical context while remaining scalable. This approach is similar to the sub-sampling also seen in representation learning for graphs and enables better scalability (Hou *et al.* 2022). ARGformer encodes paths of marginal coalescent trees and augments them with positional encodings that reflect the ordering of coalescence events and the local tree topology along the path, providing a flexible representation model for ARGs. Each node in the inferred genealogy has a unique identifier. Because marginal trees share ancestral nodes, and adjacent trees along the genome share topologies between recombination events, encoding these paths preserves coalescent and recombination history. By learning from repeated co-occurrence of ancestral nodes across samples and genomic intervals, the model can capture genealogical relationships and their evolution through time along the genome.

**Figure 1:**
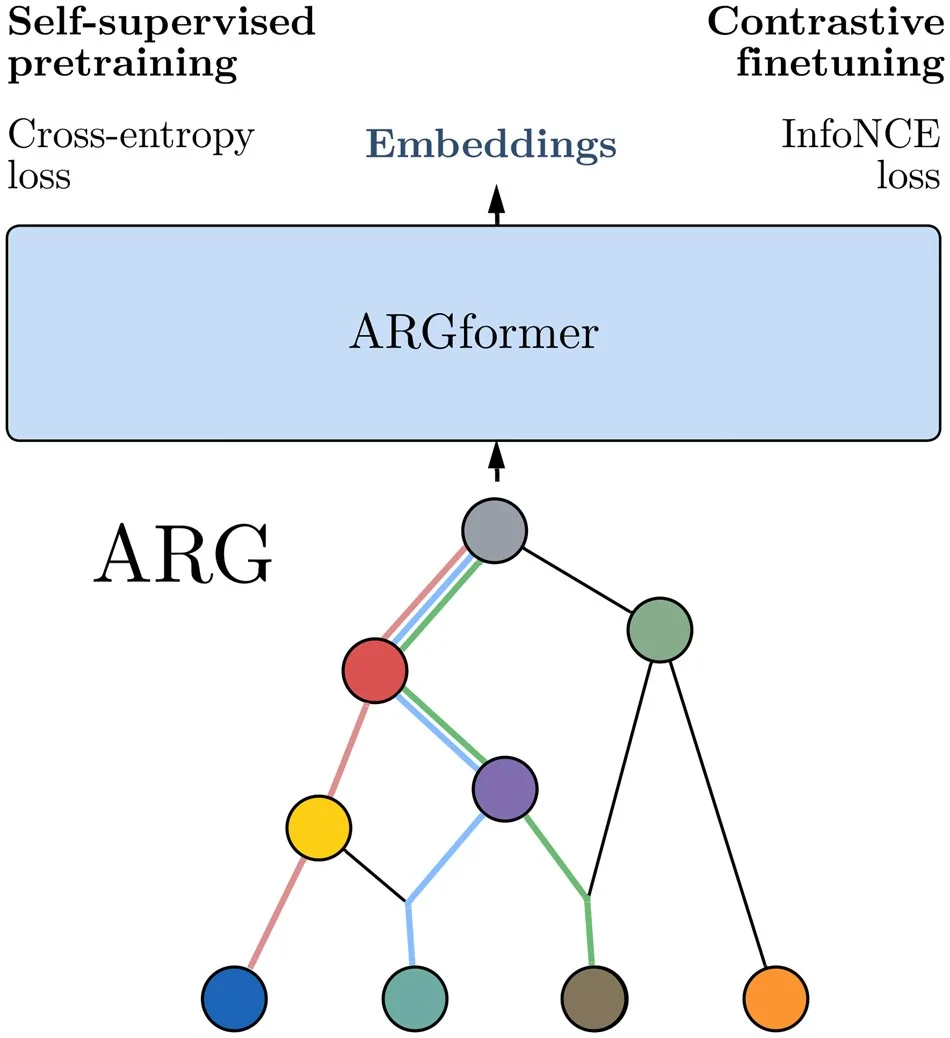
Overview of ARGformer. Schematic ARG, with colored edges showing example marginal paths, which are passed through ARGformer, an encoder-only transformer, with self-supervised masked pretraining objective, and contrastive finetuning for downstream usage of learned embeddings (visualization, clustering, retrieval for ancestry/introgression).

ARGformer is an encoder-only transformer that operates on these path sequences to learn contextual vector representations of ARG nodes (Fig. 1), which are pooled to obtain one embedding per leaf-to-root path. During self-supervised pretraining, we adopt a masked-node objective: we randomly mask node tokens along each path and optimize a cross-entropy loss to predict the masked nodes, analogous to masked language modeling but applied to the ARG. Concretely, let a leaf-to-root path be a sequence of tokens $x=(x_{1},\ldots ,x_{L})$, where each token is a node identifier. Let M⊆{1,…,L} denote the set of masked positions, sampled by independently including each position *i* in *M* with probability $p_{\text{mask}}=0.30$ (Warner *et al.* 2025). Let $\tilde{x}$ be the corrupted input sequence obtained by replacing $x_{i}$ with a special [MASK] token for all i∈M. Given the transformer hidden state $h_{i}=f_{\theta }{\left(\tilde{x}\right)}_{i}$ at position *i*, we predict the original token via a softmax head over the vocabulary


(1) $$p_{\theta }(t|\tilde{x},i)=\text{softmax}{\left(Wh_{i}\right)}_{t},\;t\in T.$$

We then minimize the masked cross-entropy loss averaged over masked positions,


(2) $$L_{\text{mask}}(\theta )=-\frac{1}{\left|M\right|}\sum_{i\in M}\;\text{log}\;p_{\theta }(t_{i}^{⋆}|\tilde{x},i),$$

Where $t_{i}^{⋆}$ is the ground-truth token at position *i*. We build ARGformer on top of a ModernBERT-style encoder that incorporates recent architectural and optimization improvements, which substantially improves training efficiency on large collections of data (Warner *et al.* 2025).

Although the resulting model after pretraining is already infused with global population structure, we finetune the pretrained encoder for downstream tasks with a supervised contrastive objective that pulls together embeddings of sequences sharing the same reference population label while pushing apart embeddings from different labels within each minibatch. Given a batch of *B* labeled sequences ${\left\{(x_{i},y_{i})\right\}}_{i=1}^{B}$ with labels $y_{i}\in \{1,\ldots ,C\}$, we encode each sequence with the transformer backbone followed by a learned pooling head, and use the unit-normalized head output as the contrastive path embedding:


(3) $$z_{i}=\frac{g_{\phi }\left(f_{\theta }(x_{i})\right)}{{\left\|g_{\phi }\left(f_{\theta }(x_{i})\right)\right\|}_{2}},$$

Where $f_{\theta }$ is the transformer encoder, $g_{\phi }$ is the pooling head, and the resulting path embeddings are normalized to unit $\ell_{2}$ norm before computing similarities. For each anchor *i*, define the in-batch positive set $P(i)=\{j\neq i:y_{j}=y_{i}\}$. We minimize the supervised contrastive (InfoNCE-style) loss (Oord *et al.* 2018)


(4) $$L_{\text{con}}(\theta ,\phi )=\frac{1}{B}\sum_{i=1}^{B}\frac{1}{\left|P(i)\right|}\sum_{j\in P(i)}\left[-\text{log}\;\frac{\;\text{exp}(z_{i}^{⊤}z_{j}/\tau )}{\sum_{k\neq i}\;\text{exp}\;(z_{i}^{⊤}z_{k}/\tau )}\right],$$

Where τ>0 is a temperature hyperparameter (default τ=0.05). The denominator sums over all in-batch samples except the anchor, providing implicit hard negatives from other classes. Anchors with no same-class partners in the batch (|P(i)|=0) are excluded from the loss. To mitigate class imbalance, we apply inverse-frequency weighting:


(5) $$L_{\text{con}}^{w}(\theta ,\phi )=\frac{1}{\sum_{i:\;|P(i)|>0}w_{y_{i}}}\sum_{i:\;|P(i)|>0}w_{y_{i}}\ell_{i},$$

Where $\ell_{i}$ denotes the per-anchor term in $L_{\text{con}}$, and $w_{c}=\frac{N}{CN_{c}}$ with *N* the total number of train examples and $N_{c}$ in class *c*.

### 2.2 ARGformer on simulated data

We first applied ARGformer to simulated data generated using msprime (Kelleher *et al.* 2016). We simulated 1600 samples from four populations (Admixed = 1000, European = 200, African = 200, East Asian = 200) in the three-population out-of-Africa demography (AmericanAdmixture 4B18) from the stdpopsim catalog (Adrion *et al.* 2020), we use the default parameters for a single 30 million base pair (bp) chromosome, using a flat recombination rate of $1\times 10^{-8}$ per bp generation and a mutation rate of 1.25e-8 per bp per generation (Browning *et al.* 2018, Lauterbur *et al.* 2023). We generated a dataset for pretraining using tskit to extract coalescent events (Wong *et al.* 2024). To better evaluate ARGformer on inferred ARGs, we inferred the ARG using tsinfer+tsdate on the simulated genotypes, without phasing or genotype error (Kelleher *et al.* 2019). We split this dataset into training (90%) and validation (10%) for pretraining.

### 2.3 Application of ARGformer to an inferred ARG of ancient and modern DNA

We applied ARGformer to real data using the inferred genealogy of ancient and present-day human genomes (Auton *et al.* 2015, Mallick *et al.* 2016, Bergström *et al.* 2020, Wohns *et al.* 2022) and constructed two task-specific datasets: one for archaic introgression analysis and one for testing Oceania-like ancestry in the Americas. Unique marginal paths were split 90/10 into train and validation sets; full population composition and dataset statistics are provided in the Supplementary Materials, available as supplementary data at *Bioinformatics* online.

## 3 Results

We first evaluated ARGformer on coalescent simulations to ask whether path embeddings recover patterns of global population structure and support ancestry-related tasks. We then applied ARGformer to ARGs inferred from ancient and present-day *Homo sapiens* genomes, using the learned embeddings to detect Denisovan ancestry in Oceania and to test for Oceanian-like affinities in Indigenous South American populations. Finally, we summarize model behavior and computational efficiency during self-supervised pretraining on large collections of marginal genealogies (see Supplementary Materials, available as supplementary data at *Bioinformatics* online).

### 3.1 Capturing population structure

**Figure 2:**
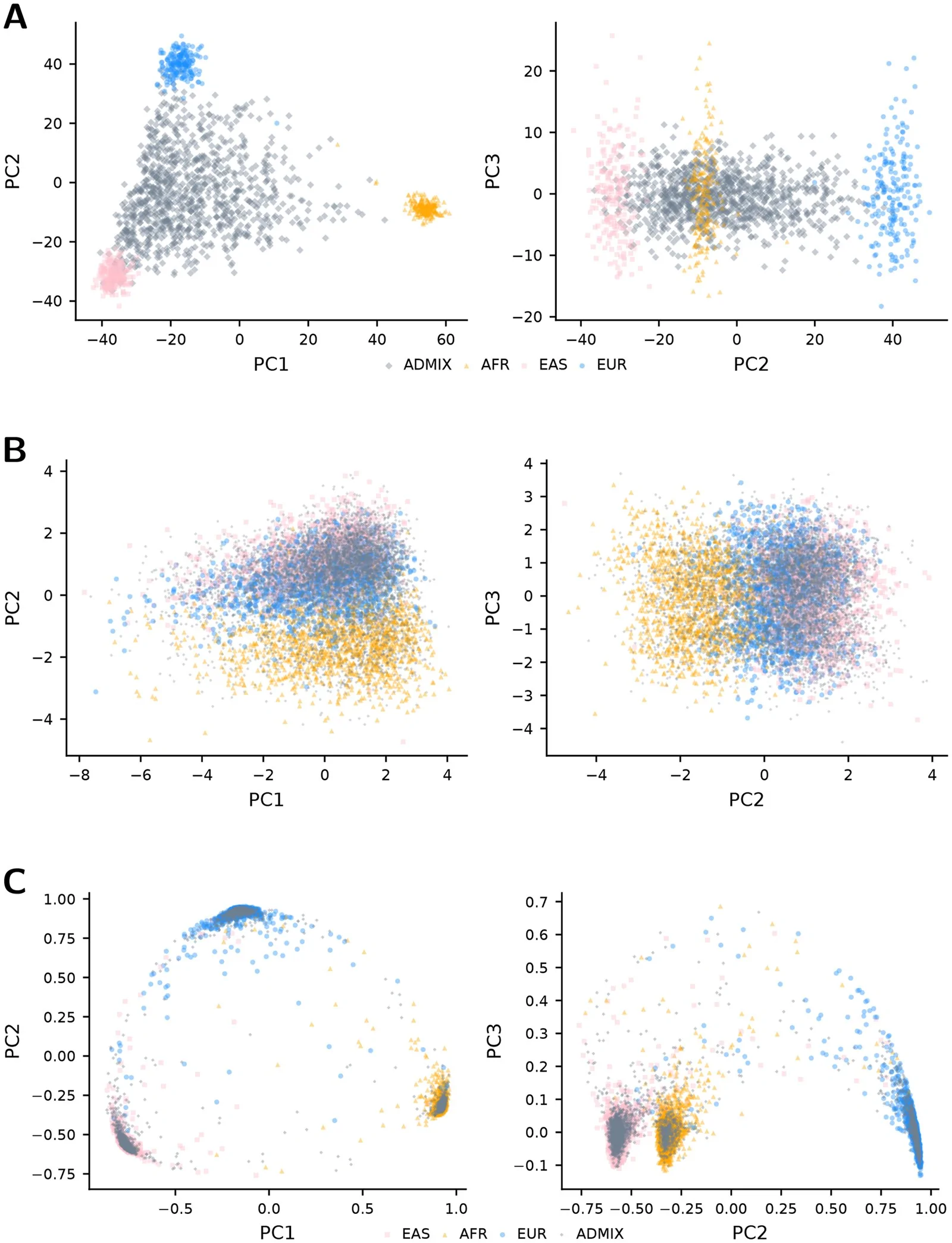
Population structure and local-ancestry geometry on simulated data. Colors indicate population label: East Asian (pink), African (orange), European (blue), and admixed (gray). (A) PCA of ground-truth genotypes (points are individuals; PC1 vs PC2; PC2 vs PC3). PCA is fitted on reference populations (AFR, EAS, EUR), and ADMIX samples are projected. (B and C) The same PCA protocol applied to ARGformer embeddings, which are generated without genotype matrices (points are haplotype marginal-path embeddings, multiple per individual). (B) shows the embeddings after self-supervised pretraining, while (C) shows the contrastive embeddings.

ARGformer embeddings provide a chromosomal segment-level view of population structure derived from local genealogies (Fig. 2). In the genotype PCA (Fig. 2A), each point aggregates information across the entire genome, so individuals with contributions from multiple ancestries will fall between the three continental clusters. In contrast, each point in Fig. 2B and C corresponds to a single marginal-path embedding, representing a local genealogy segment in the genome. Under the simulated admixture demography, local segments predominantly trace to one of the unadmixed sources and therefore concentrate within reference ancestry clusters.

To evaluate the contribution of our distinct training stages, we performed an ablation comparing the model’s representations immediately after self-supervised pretraining pooled on the extant node (Fig. 2B) to those obtained after contrastive finetuning (Fig. 2C). This comparison shows that pretraining alone already captures inherent aspects of global population structure directly from the ARG topology, prior to the introduction of any ancestry labels. Self-supervised pretraining is important because it learns a general genealogical representation from shared ancestral-node structure before contrastive training embeds held-out query lineages relative to labeled references. The subsequent contrastive finetuning then builds upon this foundation to more sharply separate the distinct local-ancestry clusters.

**Figure 3:**
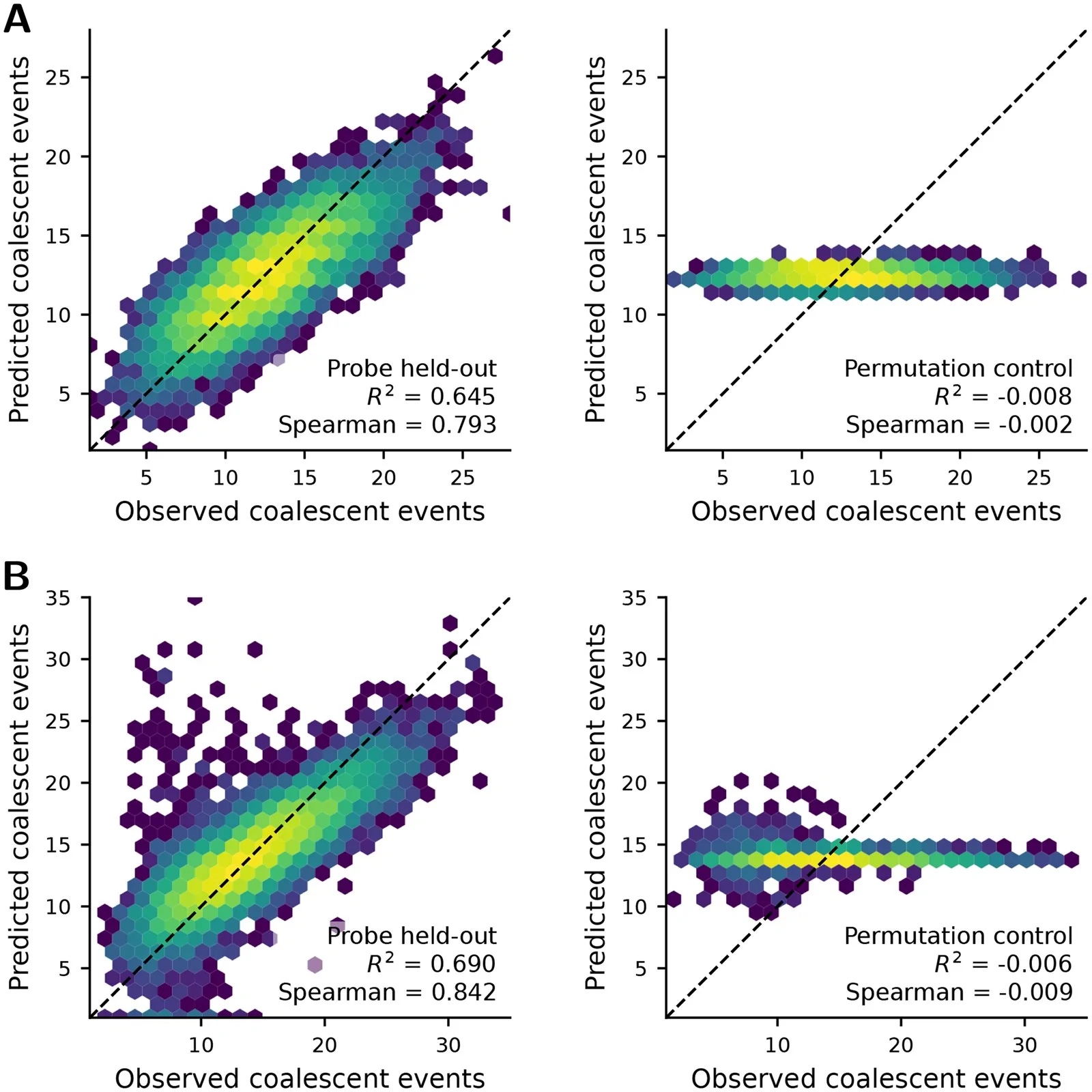
Self-supervised embeddings encode genealogical structure in both simulated and inferred ARGs. (A) American simulation. (B) Real archaic-modern inferred ARG. In each panel, the left plot shows held-out prediction of the number of coalescent events using a ridge probe on frozen embeddings, and the right plot shows the corresponding permutation control obtained by randomly shuffling the training labels before fitting the same probe.

To test whether these self-supervised embeddings encode genealogical structure rather than only downstream separability, we trained a ridge probe on frozen embeddings to predict the number of coalescent events along each path. In the American simulation, the probe achieves strong held-out agreement between predicted and observed path depth (Fig. 3A). The same analysis also generalizes to inferred genealogies from ancient and present-day humans in the archaic-modern setting (Fig. 3B), showing that the learned representation captures aspects of genealogical depth in both simulated and real ARGs. In each case, performance collapses under a permutation control in which the training labels are randomly shuffled before fitting the same ridge probe, preserving the embedding distribution and label marginal while destroying the embedding-label correspondence. This indicates that the signal is genuinely present in the learned embedding geometry. In the archaic-modern ARG, the parity plot also shows a concentration of short paths with few coalescent events, consistent with the long-branch structure of archaic lineages. Additional probe results for the Oceania-America analysis, along with attention interpretability analyses of the self-supervised model, are provided in the Supplementary Materials, available as supplementary data at *Bioinformatics* online.

Using only the learned ARG-based embeddings, we can separate local chromosomal segments without directly accessing genotype matrices. In this sense, ARGformer plays for genealogies and haplotypes a role that is analogous to PCA for genotypes, providing a low-dimensional visualization of population structure that is rooted in the inferred ARG. This contrastive structure in embedding space is what enables simple clustering and similarity-based retrieval methods to perform well on downstream ancestry classification.

We benchmarked local ancestry inference (LAI) in admixed individuals against FLARE (Browning *et al.* 2023). Because FLARE is a specialized local-ancestry method, we use it here as a task-specific baseline, while ARGformer is intended as a general representation model for multiple downstream analyses on inferred genealogies. Using $10 000$ unique marginal paths sampled from admixed individuals, we considered two inference strategies based solely on ARGformer embeddings. First, performing PCA on embeddings of unadmixed reference individuals and retaining the top 10 principal components. Second, project each path embedding into a 10-dimensional PC space and assign an ancestry label based on its cluster membership (PCA clustering).

The second strategy treats each admixed marginal path as a query and retrieves its nearest neighbors among embeddings of reference haplotypes in embedding space, assigning ancestry labels based on the labels of the retrieved neighbors. In both cases we compare the resulting three-way local ancestry calls with those from FLARE, providing a quantitative benchmark for how well the contrastively trained ARGformer embeddings capture population structure at the level of individual genomic segments. ARGformer achieved comparable accuracy to FLARE, a state-of-the-art local ancestry inference method, and obtained higher accuracy on AFR ancestry with the nearest-neighbor retrieval (Table 1).

**Table 1:** LAI precision and recall on simulated American admixture.

	EUR		EAS		AFR	
Method	Prec.	Rec.	Prec.	Rec.	Prec.	Rec.
FLARE	98.41	**98.54**	98.85	**99.74**	**99.70**	96.70
ARGformer PCA	**98.47**	97.91	98.81	99.07	97.46	97.81
ARGformer retr.	**98.47**	98.00	**98.88**	99.04	97.46	**97.90**

FLARE is run on simulated genotypes, ARGformer is run on the inferred ARG. Bold indicates the best performance, and underline indicates the second best.

### 3.2 Identifying Denisovan ancestry in Oceania

**Figure 4:**
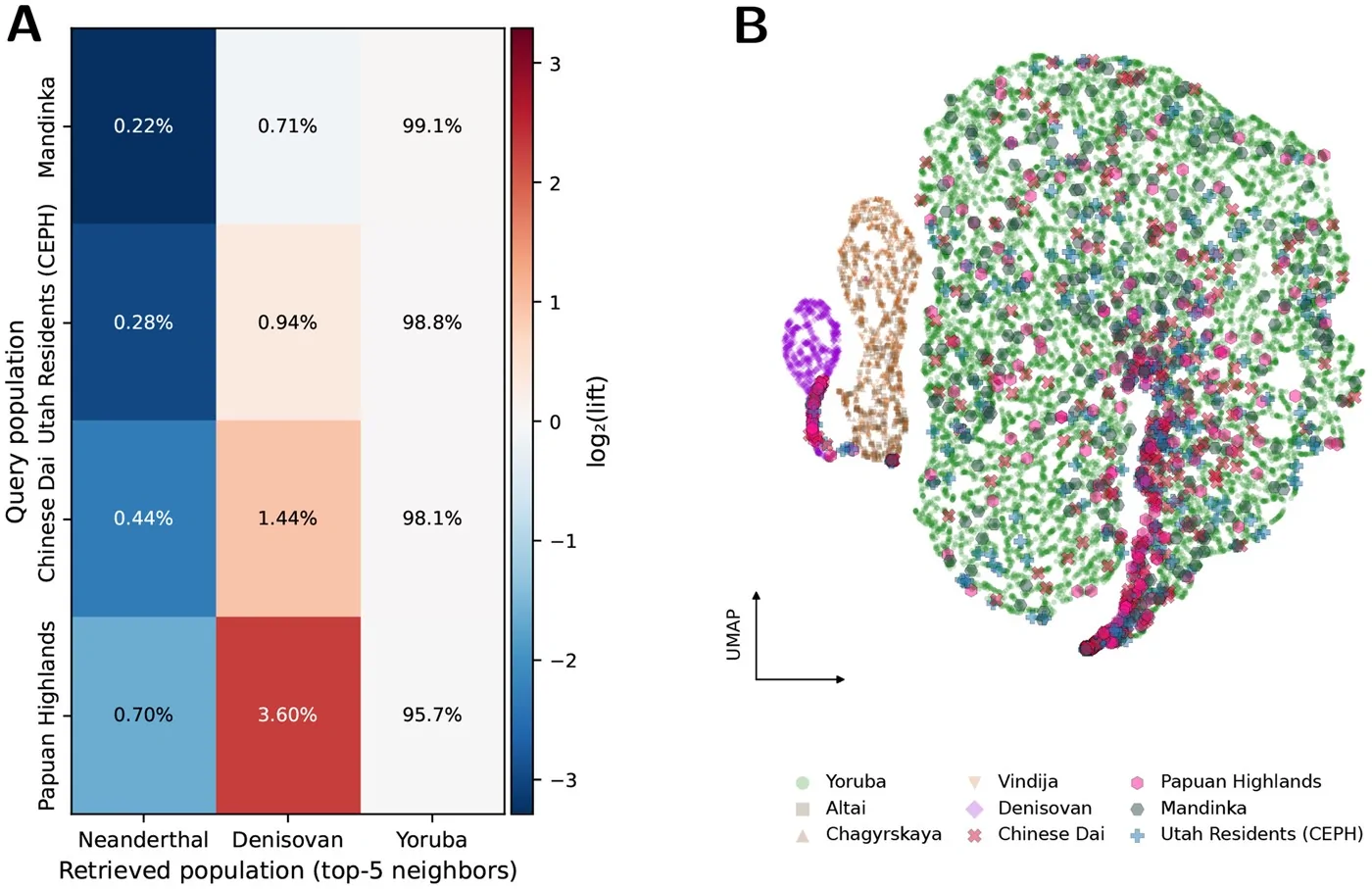
Denisovan segment retrieval in Oceania. (A) Nearest-neighbor retrieval reveals Denisovan-enriched embedding neighborhoods in Oceanian haplotypes. For each held-out query path (row), we retrieve its top-5 nearest neighbors and record the neighbors’ reference individual labels (columns). Each cell reports the row-normalized fraction of retrieved neighbors assigned to the corresponding reference population. Colors show the log-lift of retrieval relative to chance (${\;\text{log}\;}_{2}(\text{lift})={\;\text{log}\;}_{2}\left({\hat{p}}_{c}/p_{c}\right)$ where ${\hat{p}}_{c}$ is retrieved vote share and $p_{c}$ is corpus label frequency), so values above zero indicate enrichment beyond what would be expected from corpus composition. (B) UMAP projection of haplotype-path embeddings for archaic references, modern population reference, and query populations.

To test whether ARGformer embeddings capture archaic introgression signals without using genotype matrices, we framed archaic ancestry detection as a nearest-neighbor retrieval problem in embedding space. For each held-out population, we retrieved the top-5 closest haplotype-path embeddings and summarized the retrieved reference labels as a row-normalized distribution over reference populations, reporting both the raw vote share and a chance-corrected enrichment (Fig. 4).

Papuan Highlands stands out with a Denisovan neighbor fraction of 3.60% among its top-5 neighbors, significantly greater than that of the comparison populations under permutation testing (p<0.001), compared with non-Oceanian populations 1.44% (Chinese Dai), 0.94% (Utah Residents (CEPH) with Northern and Western European ancestry), and 0.71% (Mandinka) in the same analysis. This enrichment is reflected by the positive log-lift coloring in the Denisovan column (Fig. 4, Panel A), where Mandinka is the only query population with a negative lift. In contrast, all query populations predominantly retrieve Yoruba-labeled neighbors (between 95.7% and 99.1% across rows), consistent with most genomic segments being closest to modern human variation. We also note that some non-Oceanian samples (e.g. Utah Residents (CEPH) with Northern and Western European ancestry) can retrieve a slightly higher fraction of Denisovan-labeled neighbors than Neanderthal-labeled neighbors. Because the archaic genomes were computationally phased without reference panels or imputation, and because contrastive finetuning can be sensitive to such technical artifacts, we treat these retrieval shares as relative enrichment signals rather than admixture proportions.

Together, this shows that ARGformer embeddings learned from inferred genealogies can localize Denisovan-like segments in Oceanian genomes, consistent with established Denisovan introgression in Papuan-related Oceanian populations (Jacobs *et al.* 2019, Vespasiani *et al.* 2022). Panel B in Fig. 4 shows a UMAP projection, highlighting distinct clusters and a subset of query haplotypes falling on archaic clusters.

### 3.3 Oceania-like ancestry in South America

**Figure 5:**
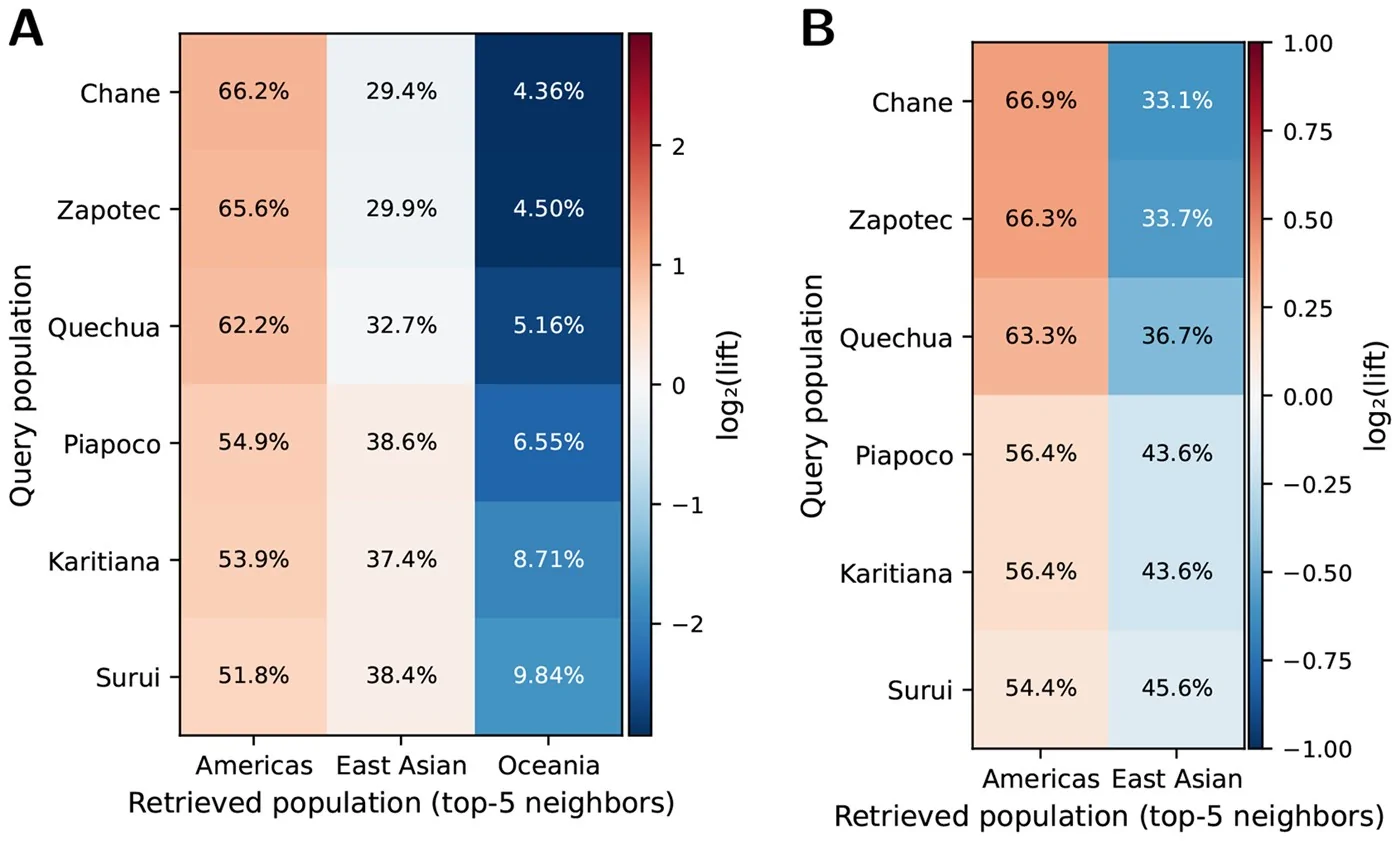
Population-scale affinities between the Americas, East Asia, and Oceania. (A) For each query population we retrieve the top-5 nearest neighbors from the embedding corpus and tabulate the neighbors’ regional labels. Suruí and Karitiana show the highest enrichment for Oceanian retrieval. (B) Negative-control retrieval with Oceanian references removed, recomputed over Americas and East Asian references only.

We next asked whether ARGformer embeddings capture the subtle Oceania-related affinities that have been previously reported in some Indigenous South American populations (Skoglund *et al.* 2015, Castro e Silva *et al.* 2021). As in the archaic introgression analysis, we framed this question as a retrieval problem in embedding space: given a query set of marginal-path embeddings from Indigenous American populations, we retrieve the top-5 nearest neighbors of its haplotype-path embeddings from a reference corpus stratified into three regional groups (Americas, East Asian, and Oceania) and summarize neighbor identities as a row-normalized vote share, alongside a chance-corrected enrichment (Fig. 5A). In this analysis, the Americas reference group comprises Pima, Maya, and Mixe individuals; the Oceania reference group comprises Australian, Bougainville, Papuan, Papuan Highlands, and Papuan Sepik; and the East Asian reference group comprises Han Chinese and Japanese.

Across query populations, retrieved neighbors at the haplotype-level are predominantly labeled as Americas, with a consistent minority labeled as East Asian, matching the expected close genealogical relationship between the Americas and East Asia under models of the peopling of the Americas. In contrast, Oceania neighbors are rare overall, but show a marked relative increase in Suruí and Karitiana. Specifically, Suruí and Karitiana retrieve Oceania-labeled neighbors at 9.84% and 8.71%, respectively. The next highest is another Amazonian group, Piapoco, at 6.55%, compared with 4.36% to 5.16% in the other Indigenous American query populations (Chane, Zapotec, Quechua). This shift is reflected in the Oceania column’s log-lift shading, where Suruí and Karitiana exhibit the weakest depletion (closest to zero) relative to the background composition of the embedding corpus, consistent with a modest but detectable excess of Oceanian-reference nearest neighbors in these populations. As a robustness check, removing the Oceanian references shifts the retrieved neighbor composition primarily toward East Asian for Suruí, Karitiana, and Piapoco, whereas the other query populations remain dominated by neighbors in the Americas (Fig. 5B).

Because each tokenized path corresponds to a marginal genealogy at a genomic interval, this enrichment can be read locally: a subset of Suruí and Karitiana segments have nearest-neighbor genealogical proximity to Oceanian reference paths that is stronger than in the other South American populations shown.

## 4 Conclusion

ARGformer leverages shared ancestral nodes to capture the topological intersections of the ARG. This allows the model to capture genealogical relationships across trees and the changes in ancestry induced by recombination along the genome. This representation serves as a flexible backbone for multiple downstream analyses, including reference-based settings in which newly threaded samples are approximately placed in an existing latent space and queried against nearby reference haplotypes. Through contrastive fine-tuning, the model learns to pull together related haplotypes and separate unrelated ones. This allows ARGformer to provide low-dimensional visualization of population structure from the ARG and to support local ancestry inference by classifying haplotypes in admixed individuals.

Together, the retrieval results support the interpretation that, although the dominant genealogical signal in South American genomes reflects shared Indigenous American ancestry with expected East Asian affinity, a subset of local genealogies in Suruí and Karitiana populations are shared with ancestry found in Oceania. Similarly, we recovered expected signals of Denisovan introgression in Oceania. Because each tokenized path corresponds to a leaf-to-root lineage, this retrieval-based view highlights how ARGformer can surface localized, ancestry-informative segments directly from inferred genealogies, without explicit genotype features, by exploiting the structure of local coalescent histories encoded in the ARG.

With appropriate architectural and objective modifications, we expect that the same framework can be extended to a broad range of other challenges in population genetics and evolutionary inference. The learned representations could potentially be adapted to graph-level tasks that detect demographic bottlenecks or signatures of selection (Browning and Browning 2015, Vaughn and Nielsen 2024). Scaling ARG-based models to biobank cohorts will likely require more compressed representations than assigning a distinct token to every ancestor. One promising direction is to define tokens that correspond to shared lineages in the genealogy, so that each individual is represented by a sequence of higher-level ancestral lineages over the past hundred or thousand generations, rather than by every individual node. Future work could also explore decoder-only or encoder-decoder architectures built on such lineage tokens for global graph-level tasks, while the present work focuses on encoder models and individual-level ancestry.

For ancestry inference, several limitations remain. Some paths in the marginal trees have few coalescent events, making them relatively uninformative and noisy for classification. A further limitation is ARG inference. Even the most scalable methods struggle with greater variant density that we expect in imputed data and whole-genomes (Gunnarsson *et al.* 2024). However, the breadth of population genetic information encoded in ARGs suggests that representation learning is a promising direction for future methods and applied analyses in population genetics.

## Supplementary Material

btag438_Supplementary_Data

## Data Availability

The data underlying this article are available in the article and in its online supplementary material. ARGformer is available at https://github.com/AI-sandbox/ARGformer.
