# Supplementary material for: ARGformer: learning on ancestral recombination graphs with transformers
Source: Bioinformatics. 2026 Aug 21;42(Suppl 2):btag438. doi: 10.1093/bioinformatics/btag438 (PMC13501305; doi:10.1093/bioinformatics/btag438)
Supplement: Supplementary file 1 [file btag438_supplementary_data.pdf]

Supplementary Material

S1. Dataset statistics

The real inferred ARG from (Wohns et al., 2022) includes samples from the 1000 Genomes Project (1KGP) (Auton et al., 2015), Simons Genome Diversity Project (SGDP) (Mallick et al., 2016), Human Genome Diversity Project (HGDP) (Bergström et al., 2020), three Neanderthal genomes (Chagyrskaya, Altai, and Vindija) (Mafessoni et al., 2020; Prüfer et al., 2014, 2017), and a Denisovan genome. We created two separate datasets for distinct analyses:

Archaic Introgression Analysis. This dataset includes archaic reference genomes (Altai, Chagyrskaya, and Vindija Neanderthals, and Denisovan), an African baseline (Yoruba), and held-out populations for validation (Utah Residents (CEPH) with Northern and Western European ancestry, Chinese Dai, Papuan Highlands, and Mandinka).

Oceania-like Ancestry in the Americas. This dataset consists of three reference groups and one test group: (1) East Asian populations (Han Chinese, Japanese), (2) Indigenous American baseline populations (Pima, Maya, Mixe), (3) Oceanian populations (Aboriginal Australian, Bougainville, Papuan populations), and a held-out test group of Indigenous American populations (Karitiana, Suruí, Chane, Piapoco, Quechua, Zapotec). Reference groups were balanced to the minimum group size to prevent biasing the model toward overrepresented populations.

We performed a 90%/10% train/validation split on the subset of unique marginal paths shown in Table S1.

Table S1. Dataset statistics for ARGformer pretraining on the real inferred ARG from (Wohns et al., 2022).

Dataset	Statistic	Training	Validation
Archaic Introgression	Sample nodes	688	
	Vocabulary size	184,963	
	Unique paths	7,660,071	851,119
	Total tokens	120,297,364	13,369,481
Oceania-like in Americas	Sample nodes	665	
	Vocabulary size	119,067	
	Unique paths	3,473,898	385,989
	Total tokens	57,777,684	6,421,781

S2. Model training and efficiency

We adopted the ModernBERT-base architecture and optimization settings (Warner et al., 2025), using a 12-layer, 12-head transformer (Vaswani et al., 2017) with rotary position embeddings (RoPE) (Su et al., 2024), and Flash Attention 2 (Dao, 2024). We assessed the model's ability to learn genealogical representations through self-supervised training. To measure generalization to unseen haplotypes, we monitored validation loss and masked token accuracy on a held-out set comprising 10% of ancestral paths excluded from training (Figure S1). On this held-out set from the inferred ARG dataset, ARGformer achieved a top-1 accuracy of 95.96%, confirming it effectively reconstructs ancestral paths from context for previously unseen sequences. Training was also highly efficient. Pretraining sustained an average throughput of 189,500 tokens/sec and converged in 58 minutes on 1 NVIDIA A100 GPU with 80 GB of VRAM.

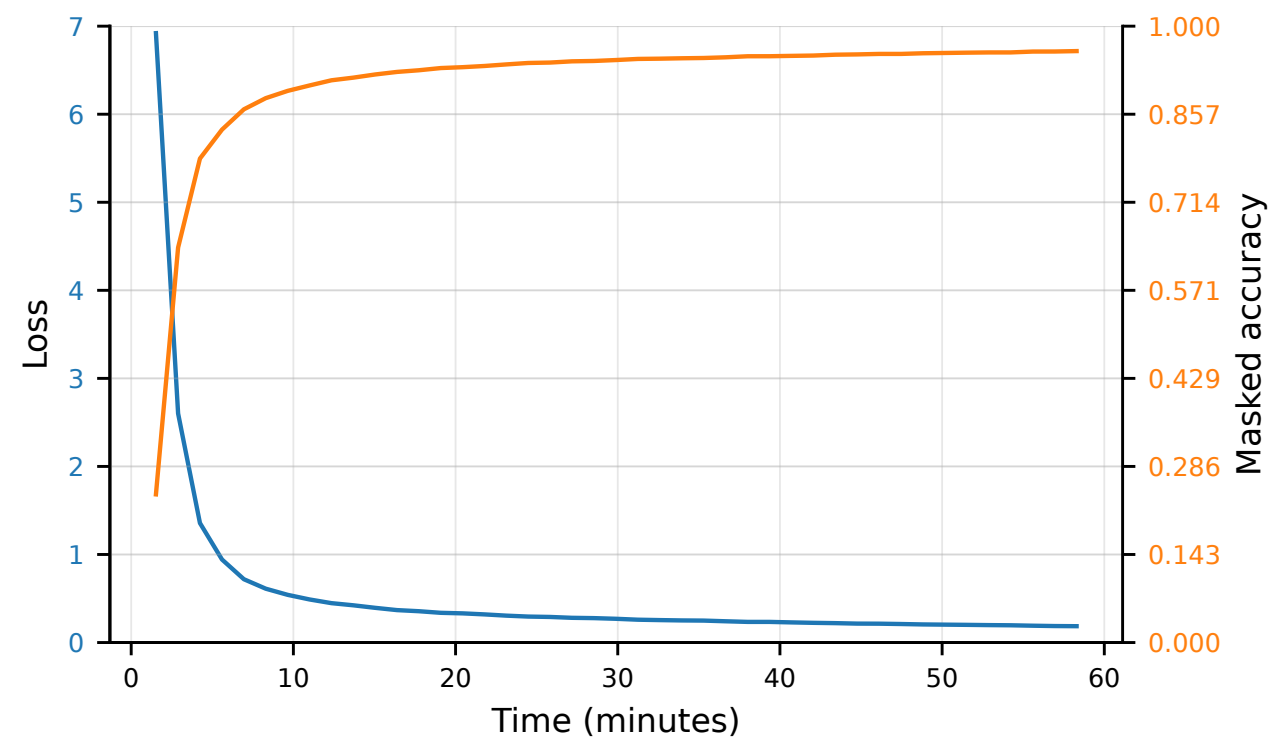

Fig. S1: Training dynamics during self-supervised pretraining of ARGformer on the inferred ARG dataset. Validation loss decreases steadily while masked-token accuracy increases over time, showing stable convergence within a single 58 minute training run on one NVIDIA A100 GPU.

S3. Visualization of Oceanian-like ancestry in South America

A complementary view comes from a UMAP projection of the haplotype-path embedding space (Figure S2). Reference population groups form their own separated clusters with most of the query points spread across the American embedding manifold, meanwhile the Oceanian cluster contains a small set of Suruí and Karitiana embeddings. A small set of Suruí and Piapoco points are also displaced toward the Oceania side of the embedding space, consistent with the retrieval asymmetry observed above.

S4. Analysis of learned representations

S4.1. Additional probing analyses of genealogical structure in self-supervised embeddings

In the manuscript we showed that a simple ridge probe trained on frozen self-supervised embeddings predicts the number of coalescent events along each path in both the American simulation and the archaic-modern inferred ARG, while collapsing under a permutation control. Here we provide an additional probe analysis for the Oceania-America inferred ARG, together with a brief summary of the probe setup.

For each dataset, we extracted frozen path embeddings from the self-supervised model and trained a ridge regressor to predict the number of coalescent events along the corresponding leaf-to-root path. Evaluation was performed on held-out paths not used to fit the probe. As a null control, we repeated the same procedure after randomly permuting the training labels, which preserves the embedding distribution, probe class, and label marginal while breaking the correspondence between embeddings and their genealogical targets.

Figure S3 shows the same analysis in the Oceania-America inferred ARG. As in the main-text settings, predictions from the probe track the observed coalescent-event counts substantially

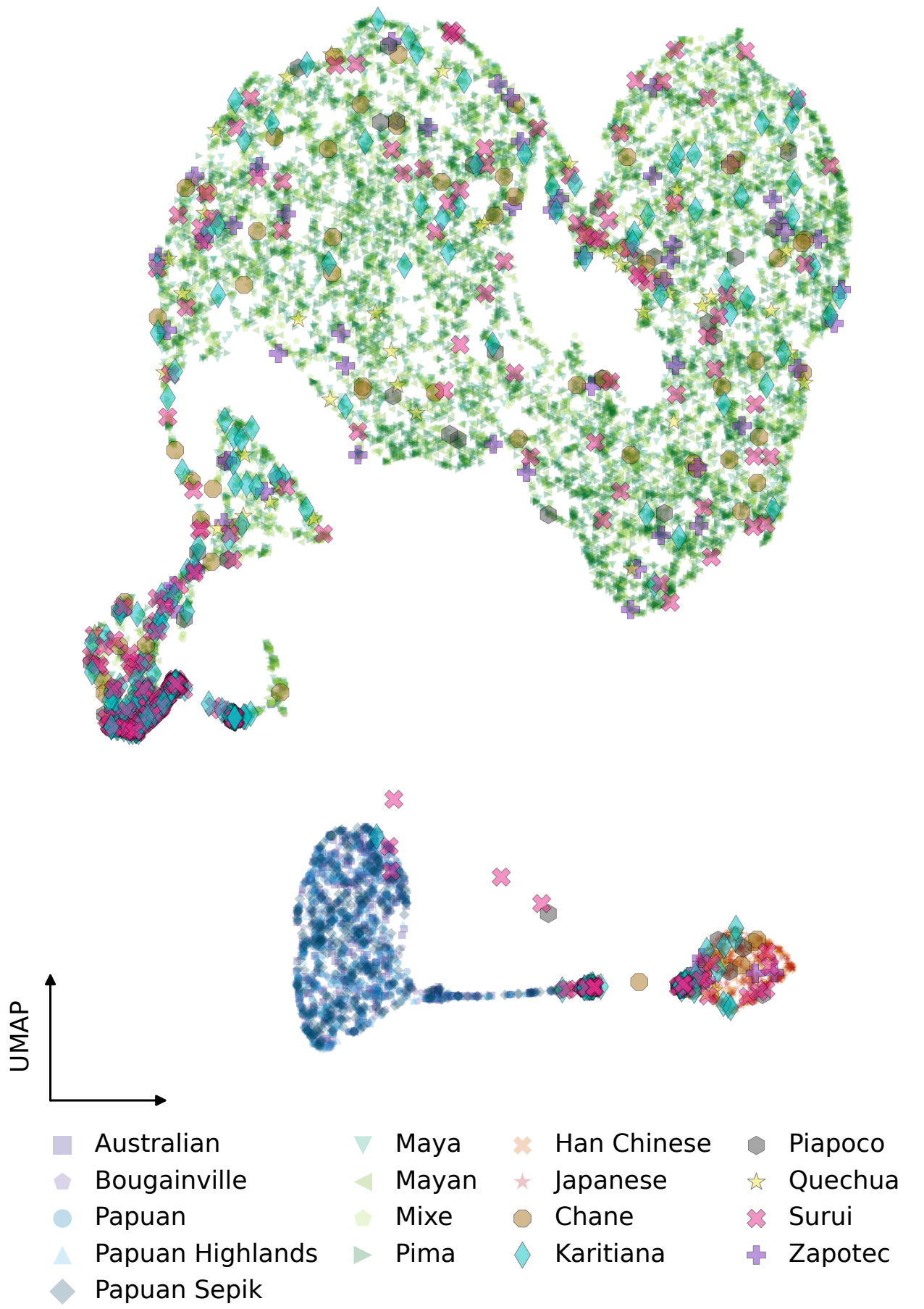

Fig. S2: UMAP projection of haplotype-path embeddings for populations spanning Oceania (e.g., Australian, Bougainville, Papuan groups), East Asia (Han Chinese, Japanese), and the Americas (e.g., Chane, Karitiana, Piapoco, Quechua, Suruí, Zapotec).

better than the permutation control. Together with the American simulation and archaic-modern results shown in the main text, this supports the conclusion that self-supervised ARGformer embeddings encode genealogically meaningful information across simulated and real inferred genealogies, and across both continental-structure and archaic-introgression settings.

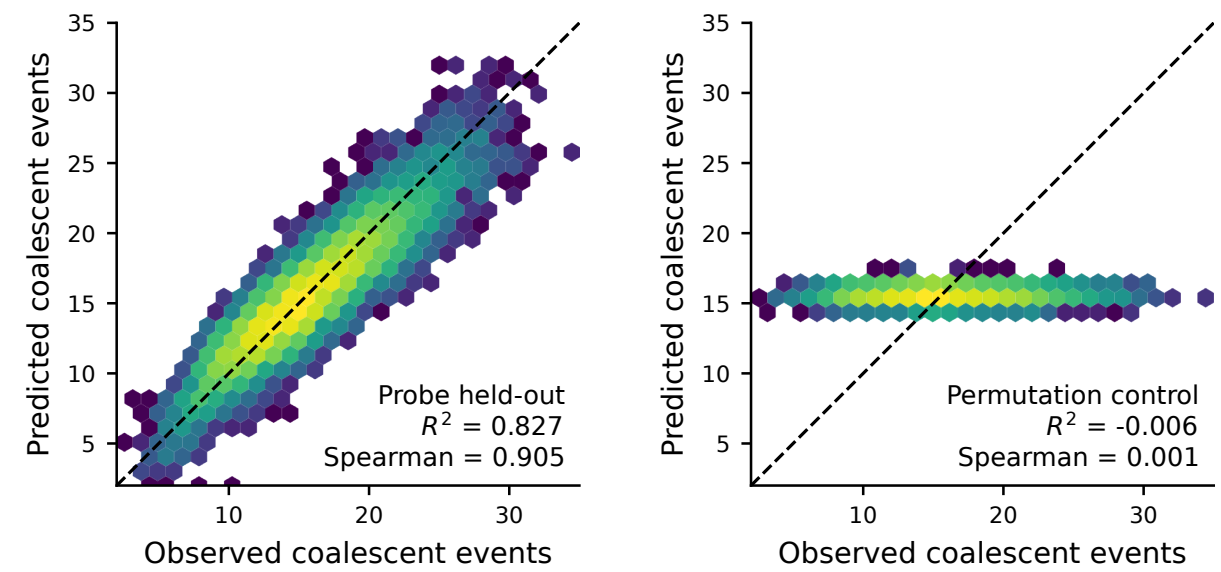

Fig. S3: Additional probe analysis for the Oceania-America inferred ARG. The left plot shows held-out prediction of the number of coalescent events along each path from frozen self-supervised embeddings using a ridge probe, and the right plot shows the corresponding permutation control obtained by shuffling the training labels before fitting. As in the main-text analyses, the probe substantially outperforms the permutation control, indicating that the self-supervised embedding space encodes genealogical depth information beyond chance.

S4.2. Attention patterns reflect structured genealogical relations

To examine what self-supervised pretraining learns beyond global embedding geometry, we analyzed node-to-node attention patterns on held-out paths from the American simulation. Figure S4 shows that different heads preferentially capture distinct genealogical relations along the leaf-to-root path. Representative examples include heads that attend to the immediate ancestor, heads that allocate attention toward the rootward end of the path, and heads that focus on deeper ancestors farther up the path.

These patterns are not uniformly distributed across the network. For each layer, we quantified each motif as the maximum fraction of node-to-node attention assigned to that pattern by any single head. The three behaviors peak in different layers, indicating that self-supervised pretraining does not simply produce a generic local-attention mechanism, but instead yields heads with partially specialized genealogical roles. Immediate-ancestor attention is strongest early and in the last layers, while deep-ancestor attention is most sustained in the middle layers and drops sharply at the end. That fits with a mid-layer role of integrating higher-level genealogical context before the final layers refocus on local prediction.

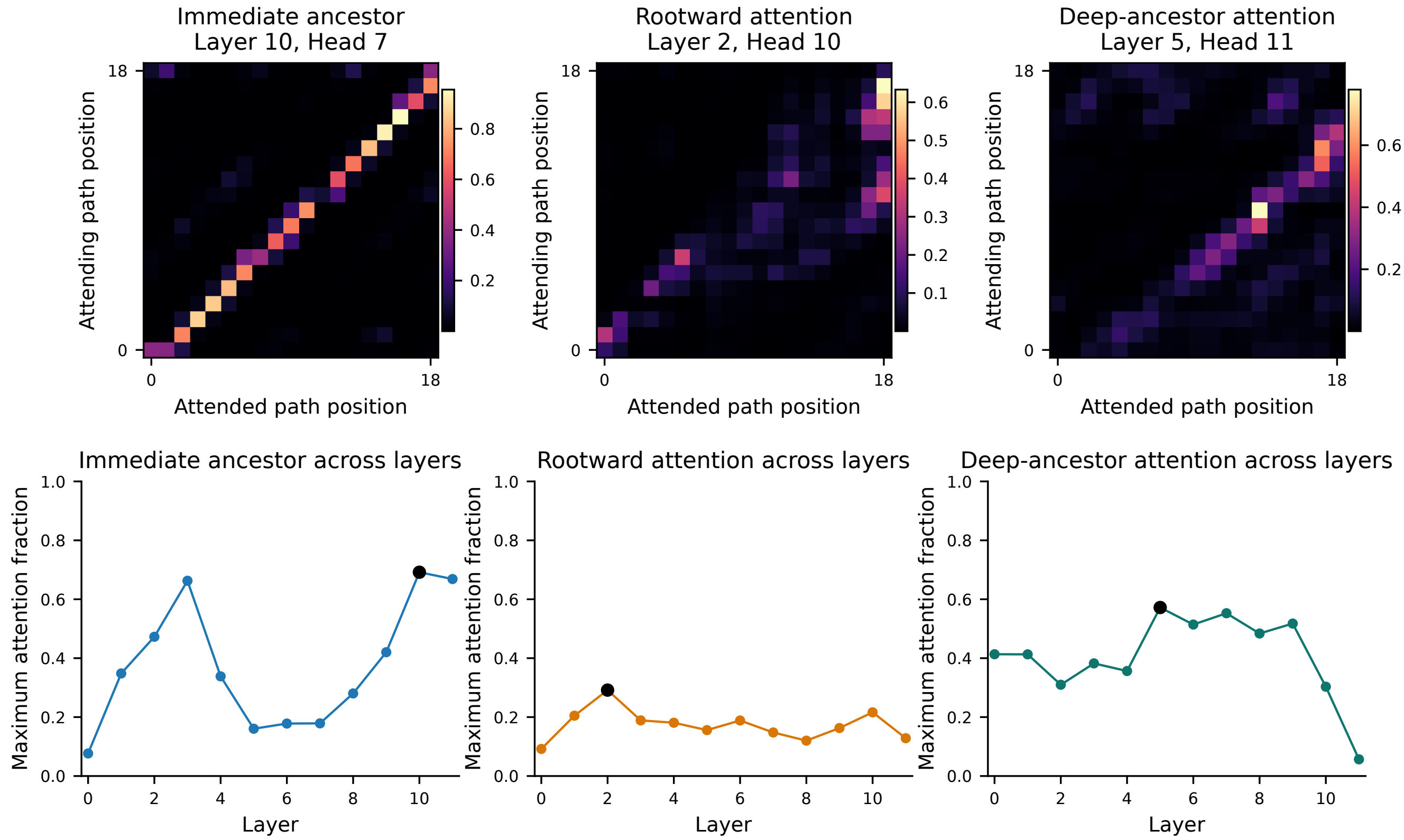

Fig. S4: Attention interpretability analysis for held-out American simulation samples. Top row: representative attention maps for heads that preferentially attend to immediate ancestor (layer 10, head 7), rootward attention (layer 2, head 10), and deep-ancestor attention (layer 5, head 11). Bottom row: layer-wise progression of each pattern, quantified as the maximum fraction of node-to-node attention assigned to that pattern by any single head in each layer. These panels show that different heads preferentially capture distinct genealogical patterns rather than all collapsing onto the same local signal.

References

A. Auton et al. A global reference for human genetic variation. *Nature*, 2015.

A. Bergström et al. Insights into human genetic variation and population history from 929 diverse genomes. *Science*, 2020.

T. Dao. Flashattention-2: Faster attention with better parallelism and work partitioning. In *ICLR*, 2024.

F. Mafessoni et al. A high-coverage Neandertal genome from Chagyrskaya Cave. *Proceedings of the National Academy of Sciences*, 2020.

S. Mallick et al. The Simons Genome Diversity Project: 300 genomes from 142 diverse populations. *Nature*, 2016.

K. Prüfer et al. The complete genome sequence of a Neanderthal from the Altai Mountains. *Nature*, 2014.

K. Prüfer et al. A high-coverage Neandertal genome from Vindija Cave in Croatia. *Science*, 2017.

J. Su et al. Roformer: Enhanced transformer with rotary position embedding. *Neurocomputing*, 2024.

A. Vaswani et al. Attention is all you need. *NeurIPS*, 2017.

B. Warner et al. Smarter, better, faster, longer: A modern bidirectional encoder for fast, memory efficient, and long context finetuning and inference. In *Proceedings of the 63rd Annual Meeting of the Association for Computational Linguistics*, 2025.

A. W. Wohns et al. A unified genealogy of modern and ancient genomes. *Science*, 2022.
